# A Quality Improvement Approach to Early Patient Discharge

**DOI:** 10.1097/pq9.0000000000000497

**Published:** 2021-12-15

**Authors:** Nicholas Stansbury, Richard Marlow Taylor, Beth Wueste

**Affiliations:** From the University of Texas Health San Antonio, San Antonio, Tex.

## Abstract

Lack of bed availability is a common problem in our pediatric unit, as in many hospitals. To address this issue, we instituted a quality improvement (QI) initiative involving collaborative nurse–physician rounding. This intervention has been shown to expedite discharge, improve patient care, and increase bed availability in other settings.

**Methods:**

By utilizing PDCA (Plan, Do, Check, Act) processes, we created two improvement initiatives, “Increasing Patient Discharge Before 12 pm” and “Midnight Rounds with Discharge Focus.” Senior resident and faculty physicians rounded on discharge-ready patients before teaching rounds, and by 10 am, placed discharge orders to allow for a 12 pm discharge. A night team consisting of senior residents and nurses conducted “Midnight Rounds” and identified potential discharges for the morning team. The project aimed to increase patient discharges before 12 pm from a June–November 2018 baseline of 15%–20% by June 2019.

**Results:**

Patient discharge percentage before 12 pm increased from 15% to 21% (*P* < 0.01) by June 2019, and as a result, bed availability increased by 16% (*P* < 0.01).

**Conclusions:**

QI methodology clarified the root causes of limited bed availability. Understanding the existing discharge process allowed for QI initiatives to develop a consistent and sustainable discharge process. Patient discharge percentages before 12 pm increased by 40%, and bed availability increased by 16% after QI implementations.

## INTRODUCTION

The lack of bed availability in hospitals is a significant issue, especially as the patient population and emergency department visits increase.^1,2^ Decreased hospital bed availability creates several problems, including decreased access to care, longer wait times, decreased patient and staff satisfaction, and diminished hospital revenue.^1,2^ Hospital bed availability could increase through the construction of new larger hospitals. However, data show that adding physical beds can exceed $1 million per bed, making physical expansion expensive.^1^ Therefore, addressing bed availability issues through improved and efficient patient flow allows hospitals to maximize their current availability while avoiding these hefty investments.

Delayed discharges significantly contribute to decreased bed availability; this problem can be improved.^1,3^ A patient’s discharge is delayed when the patient is medically ready for discharge, but they do not leave the hospital for other reasons, including the inability to place patients in appropriate post-acute care facilities, lack of supply coordination, poor communication between providers, and staffing shortages.^1,4,5^

Furthermore, late afternoon discharges create admission bottlenecks in the emergency department, leading to increases in the length of inpatient hospital stays for patients.^6^ Delay in bed access compromises patient safety, which is why the Joint Commission has urged hospitals to implement standards to manage patient flow throughout the hospital.^7–9^ Additionally, researchers in the National Health Service documented that over 1.2 million extra bed days resulted from delayed discharges, which produced over a $100 million loss annually.^10,11^

As hospital bed availability decreases and patient wait times increase, a better solution is needed to serve patients efficiently and effectively. Hospitals often choose the straightforward method of adding more hospital beds to address decreased availability issues.^7^ Adding more beds is not cost-effective and fails to address operational inefficiencies that are a root cause of decreased patient bed availability. Several studies have indicated that hospital-level interventions to expedite discharges before noon is an effective way to increase hospital bed availability.^7,12–14^

Our inpatient pediatric unit consistently reported operating with limited bed availability, which caused pediatric patients and their families to wait many hours for hospital admission as beds were unavailable. Admission delay creates a potential safety hazard for patients and a likely financial hazard to the hospital. After the Joint Commission’s hospital review, a team of medical students, residents, faculty physicians, and educational specialists acted upon their recommendations to observe and improve the discharge process.

The current literature indicates that quality improvement (QI) methodology and efficient and collaborative nurse–physician rounding can decrease the incidence of delayed patient discharges, improve patient care, and increase hospital bed availability.^15–19^ Lean Six Sigma is a process-focused QI methodology that emphasizes process optimization by removing waste and reducing variation. Healthcare institutions have adopted this QI methodology to enhance the efficiency and effectiveness of healthcare administrative and clinical processes.^20,21^

This project’s overall goal was to increase the inpatient pediatric unit’s bed availability by decreasing delayed discharges. Our specific project aim was to increase the percentage of discharges before 12 pm to 20% by June 2019 from the current baseline of 15% (June 2018–November 2018) and thus increase bed availability.

## MATERIALS AND METHODS

The Institutional Review Board of the University of Texas Health Science Center at San Antonio reviewed the study. They determined that the study was quality improvement and not human subjects research.

The QI team consisted of two medical students, a Lean Six Sigma master black belt in the pediatrics department, a faculty pediatrician, and the inpatient pediatric unit’s charge nurse.

To understand delayed patient discharges and bed availability, the QI team identified the discharge process stakeholders. The main stakeholders were faculty and resident physicians, nurses, and patients. Stakeholders were ultimately all observed at different phases during this project. Generally, there were four resident physicians and one faculty physician on the inpatient physician teams at one time. Finally, while patients are critical stakeholders, our QI team did not address their role in the discharge process.

**Fig. 1.**
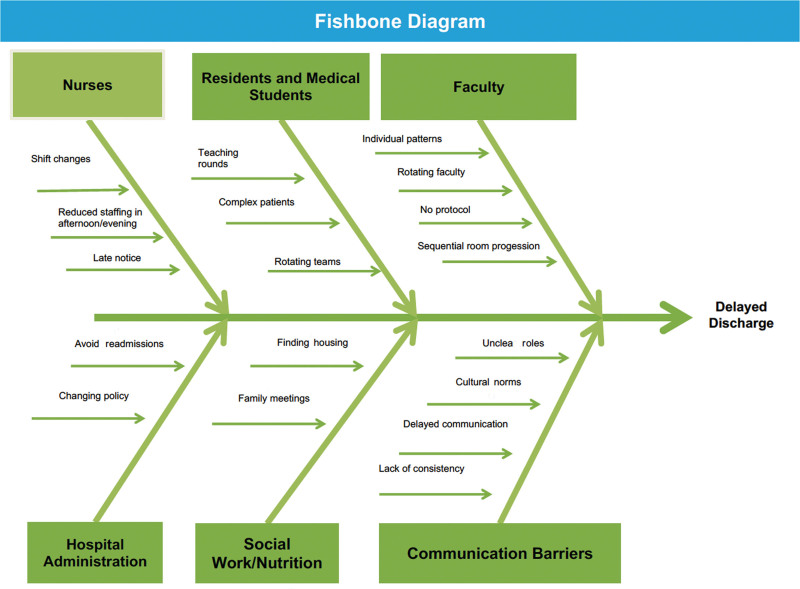
Fishbone diagram. Causes of delayed discharge.

**Fig. 2.**
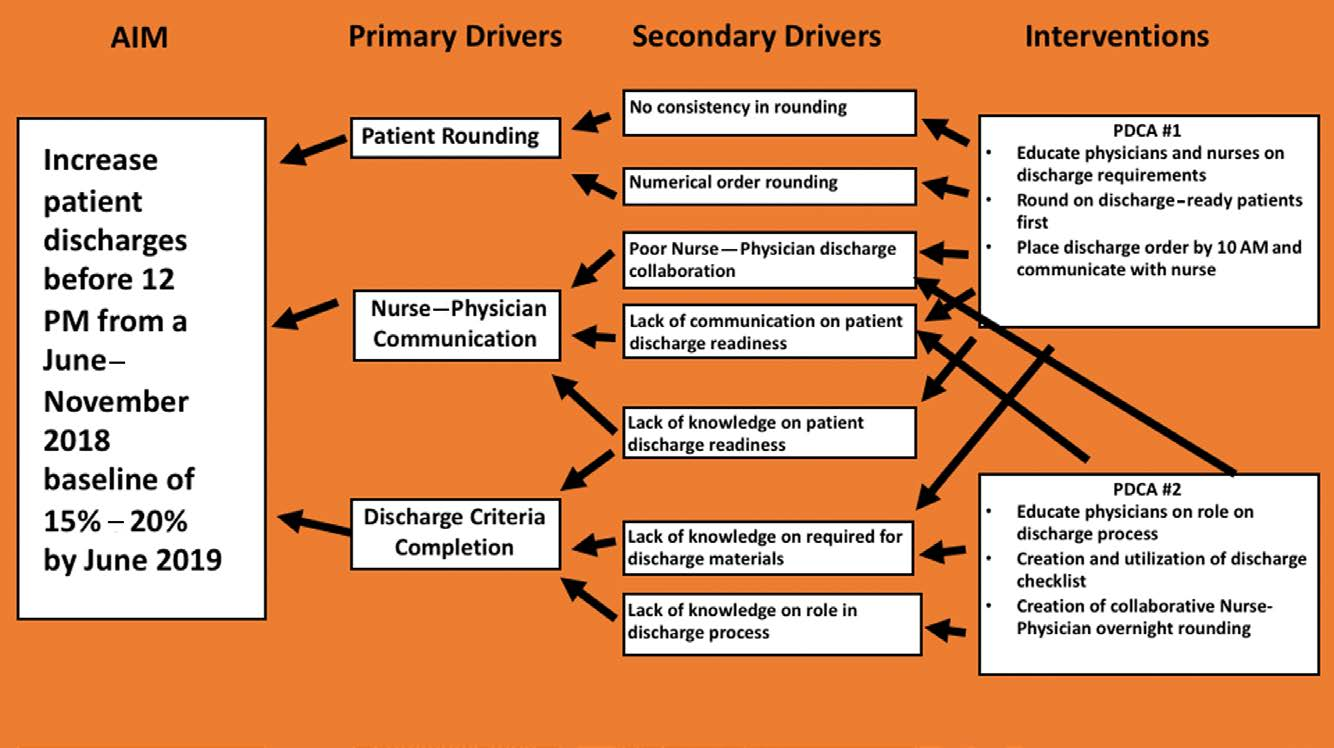
Key driver diagram.

Finally, the QI team collected baseline data via chart review on the percentage of patients discharged by 12 pm; the result was 15%. Therefore, using the baseline percentage of discharges by 12 pm and the information illuminated in the observation/interview stage, the QI team constructed a fishbone diagram of the root causes for the delays (Fig. 1) and developed a key driver diagram (Fig. 2). They created 2 PDCA cycles to address the identified root causes of delayed discharges with the project goal of increasing patient discharges before 12 pm from a June–November 2018 baseline of 15%–20% by June 2019 (Tables 1 and 2).

**Table 1.** PCDA #1: Increasing Patient Discharge Before 12 pm

*Plan*	• Data collected on average patient discharge times and percentages of discharges before 12 pm through hospital record review
	• Stakeholders interviewed about barriers contributing to delayed discharges and workflow was observed
	• Fishbone diagram (Fig. 1) created addressing delayed discharges
	• Strategy developed to round on discharge-ready patients first and write discharge orders before 10 am. The 10 am time was chosen as the nurses voiced concerns that their teams needed at least 2 h to process the discharge order during their interviews. Furthermore, studies show that discharges before 12 pm improve hospital flow by allowing for earlier admissions and increasing patient capacity.^22^
*Do*	• Senior resident and faculty physician rounded on discharge-ready patients before teaching rounds so discharge orders were placed by 10 am
	• Early discharge prioritization gave the resident physician teams time to sign and submit the discharge orders before 10 am without losing educational time
*Check*	• Evaluation performed on implementation of the *Do* action item by chart review of the percentage of patients discharged before 12 pm
	• Repeat observations preformed to ensure consistent and sustainable midnight rounds protocol implementation
*Act*	• Stakeholders informed about process changes’ success
	• Re-education preformed in areas of failure
	• New team orientation instituted procedures
	• Bimonthly reviews of bed availability data preformed

**Table 2.** PCDA #2: Midnight Rounds with Discharge Focus

***Plan***	• Nurses and physicians interviewed to understand the night-shift’s role in the discharge process
	• Midnight Rounds” to engage the night-shift in the PDCA #1 goals to prepare patients for early-morning discharge
***Do***	• “Midnight Rounds” instituted
	• Goal of “Midnight Rounds”-encourage and support the physician-nurse discussion of all patients’ status and review all items for discharge as a standard of the night shift
	• At 12 am, the senior resident physician and charge nurse met to discuss each patient’s discharge status to decrease communication delays between these two roles
	• Utilization of a discharge checklist allowed physician-nurse team to collaborate and review discharges by task to execute the necessary discharge steps overnight
	• Morning checkout physician and nurse teams reviewed the updated checklist so final discharge preparation occurred before 10 am
***Check***	• Evaluation performed on implementation of the *Do* action item by chart review of the percentage of patients discharged before 12 pm
	• Repeat observations preformed to ensure consistent and sustainable midnight rounds protocol implementation
***Act***	• Stakeholders informed about process changes’ success
	• Re-education preformed in areas of failure
	• New team orientation instituted procedures
	• Bimonthly reviews of bed availability data preformed.

In detail, the QI team evaluated the percentage of patient discharges before 12 pm using a control chart from June 2018 to June 2019 to analyze pre- and post QI initiative data. The pre-intervention phase was June–November 2018, and the post-intervention phase was December–June 2019. The data for percentages of discharges before 12 pm and the percentage of bed availability were analyzed using a student *t* test and control charts to graphically track process changes. The hospital-based quality assurance office conducted the statistical analysis.

## RESULTS

We clarified, defined, and measured the current discharge process during direct observation, noting delays in communication, gaps in patient care hand-offs, and team-based task assignments as possible problems causing patient discharge delays as outlined in Figure 1. After the observation phase, a key driver diagram (Fig. 2) illuminated the primary and secondary drivers that contributed directly to achieving the project aim and allowed the creation of PDCA #1 and #2.

After implementation of PDCA #1 and #2, the percentage of patients discharged before 12 pm increased from 15% to 21%, t(12) = t-value = -6.41, *P* < 0.01 from the pre-intervention phase June 2018–November 2018 to the post-intervention phase December 2019–June 2019. This improvement was an approximately 40% increase in discharges before 12 pm.

Additionally, unit bed availability increased by 16% t(12), t-value= -9.04, *P* < 0.01 from the pre-intervention phase June 2018–November 2018 to the post-intervention phase December 2019–June 2019.

**Fig. 3.**
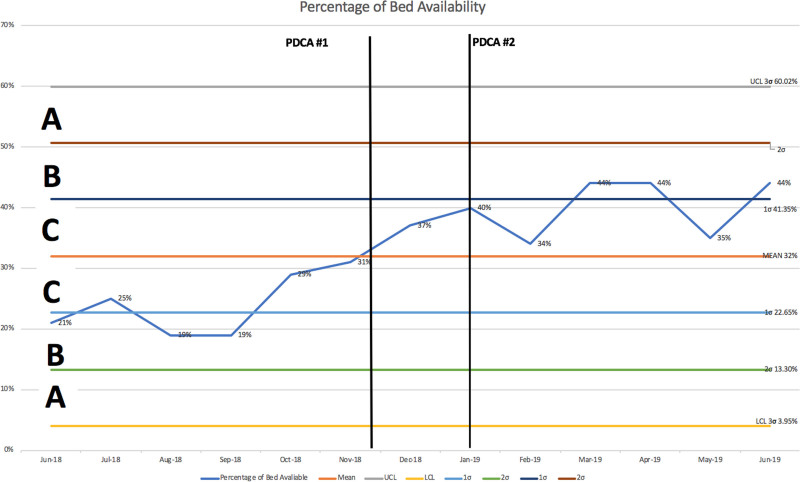
Percentage of discharges before noon.

**Fig. 4.**
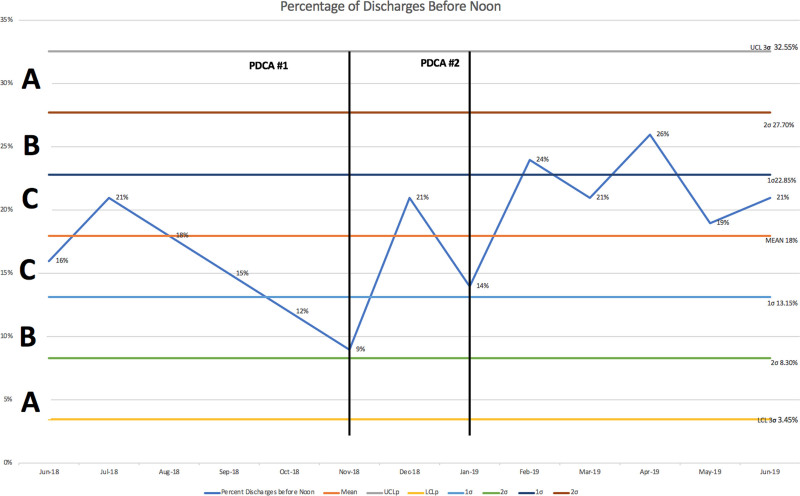
Percentage of bed availability.

Figures 3 and 4 outline the control charts for percentage of discharges before noon and percentage of bed availability, respectively. There are total of 13 data points with 6 data points in the pre-intervention phase and 7 data points in the post-intervention phase. In Figures 3 and 4 through zone testing and upper and lower control limits, the data demonstrate to be within control during the entire testing process. While a true trend was not seen in either Figure 3 or 4, Figure 3 demonstrates a positive shift with 6 or the 7 data points after the intervention above the mean compared with 5 of the 6 data points at or below the mean before the intervention. Figure 4 demonstrates a positive shift with all 7 data points after the intervention above the mean and 3 of the 7 data points greater than 1 SD above the mean compared with all 6 data points at or below the mean before the intervention with half of the data points greater than 1 SD below the mean.

## DISCUSSION

After identifying stakeholders, we performed in-depth interviews to understand the barriers contributing to delayed discharges. After evaluating the current inpatient pediatric ward bed availability, the team of stakeholders observed and evaluated each phase of patient care from admission to discharge. Next, we observed morning meetings between nurses and resident physicians, physician rounds, and shift change handoffs. These observational data points allowed the team to identify potential non-value-added steps in the discharge process to illuminate the root causes of delayed discharges.

As our team embarked on the QI initiatives to increase bed availability, the observation phases revealed that the inpatient pediatric unit’s overall discharge process lacked consistency and efficiency that led to delayed communication between nurse–physician teams, night shift and day shift gaps in the hand-off of patient care, and unclear expectations of roles and tasks required to facilitate early discharges as outlined in Figure 1.

Observations showed that each faculty physician rounded individually without a consistent rounding protocol and often according to numerical room order. The problem with numerical room rounding is not immediately obvious, but it affects discharge times. Patients ready for discharge at the bottom of the patient list were not rounded upon until the end of lengthy teaching rounds, which subsequently delayed discharge orders until mid-afternoon since the focus of rounding at an academic institution is advanced education. This practice left some patients not discharged until 5 pm. In addition to faculty variation, new resident teams rotated through the unit every few weeks, perpetuating team inconsistency and leading to a steep learning curve. They attempted to build nurse–physician relationships and learn service-based needs without standard protocols or consistent role modeling. Lastly, there was no established guidance to know when to place discharge orders and no consistent messaging on why early discharge was significant. Ultimately this lack of protocol left patients waiting until late in the day to be released from the unit.

Even after the team performed observations and interventions based on PDCA cycles showing improvement (Table 1), we noted that many residents and faculty physicians were still unaware of the PDCA’s new discharge protocol. The nature of resident training involves a constant rotation of different resident and faculty physicians working on various services. These constant personnel changes and poor handover communication caused setbacks because many stakeholders were unaware of the project from month to month. However, multidisciplinary buy-in increased after the QI team performed a combined re-education of all physicians, residents, and nurse leaders. In addition, the team placed visual rounding instructions on team workrooms and nurse stations to reinforce new protocols. All of these subsequent interventions were key to establishing a robust control plan to carry the work forward. Still, one area that the initial re-education could not address was the night team’s view of themselves as only interval management. This perception led to creating the second quality initiative to determine how best to support a collaborative system and sense of responsibility between the night shift residents and nurses to complete initial orders and necessary discharge items to prepare patients overnight for a morning discharge.

The night team became an essential part of management and planning to increase the focus on discharge in the inpatient pediatric unit. During the day shift, the senior resident physician met with the charge nurse and discussed patients with likely clear discharge criteria. This conversation allowed nurses time to prepare discharge action items for these patients. However, before this project’s intervention, we realized that these discussions only took place once every 24-hours and only during the day shift. If we were to make the entire inpatient pediatric unit discharge focused, we had to involve nurses with the night team. We initiated another PDCA cycle to carry forward our long-term control plan, which ultimately established “Midnight Rounds.”

During “Midnight Rounds,” the senior resident physician and the charge nurse on the night shift reviewed discharge items and status on every patient in the inpatient unit. Resident physicians and nurses often participate in separate role-specific shift handoffs when the day team and night team switched; therefore, collaborative crosstalk between physicians and nurses during “Midnight Rounds” often resulted in incomplete information-sharing regarding patient discharge timelines. Having senior residents and charge nurses meet during the night, each role could set up patients for discharge more effectively and have comprehensive information to communicate between roles during hand-offs. Physician–nurse collaborative and shared planning corrected gaps in the discharge process before the morning arrived, allowing day-time discharges to proceed with fewer delays.

Overall, the project surpassed the initial goal by one-third by increasing discharges before 12 pm from 15% to 21% (40%) and increasing available beds by 16%. While we did not attempt to directly measure this improvement in terms of increased revenue to the hospital or overall improved patient satisfaction, seeing that the results allowed for increased bed availability and earlier discharges, increased hospital revenue and patient satisfaction are logical effects.

Overall, these quality initiatives demonstrate a remarkable ability to improve discharge times and increase bed availability by focusing on consistency, clear communication, and collaboration between nurses and physicians. We overcame a significant hurdle in the traditional medical practice of numerical order by instituting a discharge-focused, consistent rounding protocol. Overall, this study supports the concept that hospitals can increase bed availability by reducing delayed discharges by utilizing a collaborative night shift to conduct “Midnight-Rounds” to plan for next-day discharges before 12 pm. Using methods outlined by QI methodology and referencing the current literature, we found the only reliable way to break these non-value-added steps was to get buy-in from champions at every level with small wins that encourage greater buy-in as the process gained stability.

Finally, an unexpected significance found in this project was the realization that physicians at all levels of education, training, and practice are expected to add value to hospital processes through quality conscience leadership. Yet, at no point in their educational journey are they provided with a formal curriculum on how to accomplish this organizational goal. This lack of understanding of making quality improvements inside a complex system leads providers to create “workarounds” that are temporarily effective and not sustainable. This project shows that training in Lean Six Sigma methodology combined with a self-directed learning project in the early years of medical education may lead to informed practices by early-career physicians. This value-added knowledge offers physicians the understanding of multi-tiered process reviews that can lead to team-based sustainable improvement practices aligned with organizational expectations and goals.

### Limitations

This study contained several limitations. First, we conducted our study at a single pediatric inpatient unit of an academic hospital. However, the QI methods used were not specific to pediatric services or academic institutions. Therefore, we believe the methods and results presented are generalizable to most hospital services. Nevertheless, further research should be conducted in various services and hospital types to confirm results and methodology. Second, the study involved constantly rotating teams due to the nature of residency training. This continued alteration may have led to variation in data and project success. Therefore, future research needs to examine rotating teams and nonrotating teams to analyze if this rotation affects data. Third, this is one of only a few studies that instituted “Midnight Rounds” to decrease delayed discharges. Further research is required to evaluate if this was a site-specific success or generalizable to other institutions.

## CONCLUSIONS

The percentage of patients discharged before 12 pm has increased by 40%, and bed availability increased by 16%. Using a QI approach, we made discharge communication and collaboration a top priority, replaced numerical order rounding with discharge-ready rounding, and instituted “Midnight Rounds,” which allowed the night team to change their role from patient monitoring to patient management and become an essential part of the discharge management team.

## DISCLOSURE

The authors have no financial interest in relation to the content of this article.
